# A cellular senescence-related genes model allows for prognosis and treatment stratification of hepatocellular carcinoma: A bioinformatics analysis and experimental verification

**DOI:** 10.3389/fgene.2022.1099148

**Published:** 2023-01-12

**Authors:** Jiaming Li, Rongzhi Tan, Jie Wu, Wenjie Guo, Yupeng Wang, Guoxing You, Yuting Zhang, Zhiyong Yu, Yan Geng, Jie Zan, Jianfen Su

**Affiliations:** ^1^ School of Biomedical and Pharmaceutical Sciences, Guangdong University of Technology, Guangzhou, China; ^2^ Department of Pharmacy, Guangzhou Panyu Central Hospital, Guangzhou, China; ^3^ The Second School of Clinical Medicine, Southern Medical University, Guangzhou, Guangdong, China

**Keywords:** hepatocellular carcinoma, cellular senescence, stratified model, precision medication, immune checkpoint, clinical treatment

## Abstract

**Introduction:** Hepatocellular carcinoma (HCC) is the most common type of primary liver cancer with low 5-year survival rate. Cellular senescence, characterized by permanent and irreversible cell proliferation arrest, plays an important role in tumorigenesis and development. This study aims to develop a cellular senescence-based stratified model, and a multivariable-based nomogram for guiding clinical therapy for HCC.

**Materials and methods:** The mRNAs expression data of HCC patients and cellular senescence-related genes were obtained from TCGA and CellAge database, respectively. Through multiple analysis, a four cellular senescence-related genes-based prognostic stratified model was constructed and its predictive performance was validated through various methods. Then, a nomogram based on the model was constructed and HCC patients stratified by the model were analyzed for tumor mutation burden, tumor microenvironment, immune infiltration, drug sensitivity and immune checkpoint. Functional enrichment analysis was performed to explore potential biological pathways. Finally, we verified this model by siRNA transfection, scratch assay and Transwell Assay.

**Results:** We established an cellular senescence-related genes-based stratified model, and a multivariable-based nomogram, which could accurately predict the prognosis of HCC patients in the ICGC database. The low and high risk score HCC patients stratified by the model showed different tumor mutation burden, tumor microenvironment, immune infiltration, drug sensitivity and immune checkpoint expressions. Functional enrichment analysis suggested several biological pathways related to the process and prognosis of HCC. Scratch assay and transwell assay indicated the promotion effects of the four cellular senescence-related genes (EZH2, G6PD, CBX8, and NDRG1) on the migraiton and invasion of HCC.

**Conclusion:** We established a cellular senescence-based stratified model, and a multivariable-based nomogram, which could predict the survival of HCC patients and guide clinical treatment.

## 1 Introduction

Hepatocellular carcinoma (HCC) is the most common type of primary liver cancer, accounting for about 75%–80% of liver cancer. In 2018, the number of new cases of liver cancer worldwide reached 840,000, ranking seventh among all malignant tumors (Bray et al., 2018). At present, the clinical treatment of HCC is mainly surgery, combined with interventional therapy, radiotherapy, and chemotherapy, targeted drugs and other treatment methods (Glantzounis et al., 2022). Despite immunotherapy for HCC patients has made great progress (Hu et al., 2022; Zhu et al., 2022), HCC patients still have a high recurrence and metastasis rate and low 5-year survival rate (Miyata et al., 2022; Reveron-Thornton et al., 2022). Thus, establishment of a precise classification method for HCC based on the clinical characteristics, immune infiltration, and sensitivity of chemotherapeutic drugs may provide reference for precision medication in the clinic and improve the clinical outcomes.

Cellular senescence is characterized by permanent and irreversible cell proliferation arrest, which occurs in response to some endogenous or exogenous stimuli including telomere dysfunction, oncogene activation (Leal et al., 2008). Cellular senescence plays an important role in tumorigenesis and development (Schmitt et al., 2022). On one hand, cellular senescence ensures tissue homeostasis and prevents tumorigenesis and proliferation in situations where senescent cells enter permanent cell cycle arrest (Nardella et al., 2011). On the other hand, senescent cells release senescence-associated secretory phenotype (SASP), including interleukin-6 (IL-6), IL-8, and matrix metalloproteases (MMPs), which promotes tumor development (Coppé et al., 2008; Okamura et al., 2022). Recent studies report that induction of senescence in liver cancer cells is a potential therapeutic approach (Wang et al., 2022). Thus, clarifying the role of cellular senescence in HCC development is beneficial for precision medication.

In this study, we analyzed the differentially expressed genes (DEGs) involved in celluar senescence between normal and HCC specimens and constructed the prognostic risk score signature of cell senescence. This signature could predict the malignant degree and prognosis of HCC patients and effectively guide clinical chemotherapy. The results of this study may provide a new strategy for exploring the treatment of HCC.

## 2 Materials and methods

### 2.1 Data collection

RNA-seq data from HCC patients, including 369 tumors and 50 normal samples, were collected from the Cancer Genome Atlas (TCGA) database (http://portal.gdc.cancer.gov/). Based on patient’s ID, patients’ clinical data were compared with their transcriptome data, which were screened using the following inclusion criteria: 1) histological diagnosis of HCC, 2) available expression profile, and 3) available suvival data. Data that met inclusion criteria were extracted from the TCGA-LIHC dataset (344 patients) for subsequent analyses. The validation set included 332 HCC patients from the International Cancer Genome Consortium (ICGC) -LIRI-JP data set (https://dcc.icgc.org/releases/current/Projects/LIRI-JP). Cellular senescence-related genes were obtained from CellAge data set (https://genomics.senescence.info/cells/).

### 2.2 Identification of cellular senescence-related DEGs (CSGs)

The “DESeq2” package (Love et al., 2014) was used to identify differentially expressed genes in 369 tumors and 50 adjacent normal samples by analyzing the count data of mrna expression and the intersection of DEG and cellular senescence-related genes was took to get CSGs. Adj. *p*-value < .05 and | log2 FoldChange (FC) | > 1 was set as cut-off value. The “ggplot2” package was used to map the venn diagrams for differentially expressed genes and celluar senescence-related genes. The “ggpubr” package was used for visualization of differentially expressed genes.

### 2.3 Construction of a prognostic model for CSGs

TCGA dataset (*n* = 344) were used as the training set. Then, univariate Cox regression was used to screen CSGs (associated with the overall survival) (*p*-value < .05) in the training set. Subsequently, a prognostic risk model was established in HCC through the minimum absolute shrinkage and selection operator (LASSO) regression. Risk score = Ʃ (βi*Expi), where βi represented the corresponding regression coefficients of each candidate prognostic gene, and Expi was the candidate gene’s expression value. We divided the training set into two groups: high risk group and low risk group based on the median riskscore of the trainingset. ICGC dataset was used as a validation set. To assess the predictive capacity, Kaplan-Meier (K-M) survival curves were examined using the “survival” and “survminer” packages, and receiver operating characteristic (ROC) curves were created using the “timeROC” package. In addition, the R package “RMS” was used to construct nomogram models linking characteristic risk scores. Clinical factors, and calibration curves were used to evaluate the models. The University of ALabama at Birmingham CANcer data analysis Portal (UALCAN) (http://ualcan.path.uab.edu/) was uesed to analyze the mrna and protein expression levels of four CSG in tumor and normal samples. Gene Expression Profiling Interactive Analysis (GEPIA) (http://gepia.cancer-pku.cn/) was uesed to analyze with the overall survival curves of according four CSGs expression. Human Protein Atlas (HPA) (https://www.proteinatlas.org/) obtain the immunohistochemistry of four genes in both HCC and normal samples.

### 2.4 Analysis of tumor microenvironment and immune checkpoints

Single-sample Gene Set Enrichment Analysis (ssGSEA) was used to calculate the scores for 28 immune cell types by the “GSVA” package (Hänzelmann et al., 2013). The ImmuneScore, StromalScore, and ESTIMATEScore were calculated using the ESTIMATE algorithm through “ESTIMATE” R package (Yoshihara et al., 2013). The immune checkpoint activation between high- and low-risk groups was also examined by the “ggpubr” package. The “pRRophetic” was used to estimate drug sensitivity. Fifty percent of cellular growth inhibition (IC50) was used as an indicator of drug sensitivity.

### 2.5 Functional enrichment analysis

The “DEseq2” package was used to identify differential genes in high-risk and low-risk groups by analyzing count data of mRNA expression. Adj. *p*-value < .05 and | log2 FoldChange (FC) | > 1 was set as cut-off value. Gene Ontology (GO) and Kyoto Encyclopedia of Genes and Genomes (KEGG) analysis of the significantly upregulated and downregulated DEGs were analyzed by the “clusterProfiler” package (Yu et al., 2012), which were used to compare biological subject among gene clusters. The GO and KEGG analyses associated with adjusted *p*-value <.05 were considered to be statistically significant.

### 2.6 Identification of hub genes in regulation network

STRING (https://string-db.org/) is a biological network database of protein interactions. The protein-protein interaction (PPI) of DEGs-encoded proteins was demonstrated by STRING (version 11.0), and a score >.4 with high confidence interaction was as significant value, and the unconnected nodes in the network were hidden. PPI network construction was conducted by Cytoscape (version 3.8.2). Plug-in CytoHubba was used to identify hub genes and sub-networks from complex interaction group.

### 2.7 Cell culture

The cell lines present in this study were obtained from the Procell Life Science & Technology Co., Ltd (Wuhan, China). Hepatocellular carcinoma line SMMC-7721 cells were cultured in high glucose-containing DMEM supplemented with 10% fetal bovine serum in 95% humidified air and 5% CO_2_ at 37°C. Small interfering RNA against EZH2 (si-EZH2, GAA UGC CCU UGG UCA AUA U), G6PD (si-G6PD, GCU CUG ACC GGC UGU CCA A), CBX8 (si-CBX8, ACG GAC GUG ACC UCA AAC UUU) and NDRG1 (si-NDRG1, GCC UAC AUC CUA ACU CGA UUU) and their negative control (scramble, UUC UCC GAA CGU GUC ACG U) were purchased from RiboBio Co., Ltd (Guangzhou, China).

### 2.8 Scratch wound healing assay

SMMC-7721 cells were evenly planted in a 24-well plate with 4 × 10^5^ cells per well. The plate was vertically scratched with a 200 μL sterile pipette tip when the cells covered 90% of the plate bottom area. After that, the culture medium in the plate was discarded and gently washed with PBS for three times, and the cell debris residue was rinsed off to make sure the visual field clear during photographing. The culture medium containing 1% FBS was added to the 24-well plate. A 3 mm wound was introduced across the diameter of each plate. Cell migration was observed by microscopy at 24 h.

### 2.9 Transwell assay

Cells in logarithmic growth phase were seeded at the upper transwell chamber insert at a density of 3 × 10^4^ cells per well. The chamber was placed in a 24-well plate in which the upper chamber contained serum-free cell culture medium and the lower chamber contained 20% FBS complete medium. The culture was continued for 24 h. The medium was discarded, and stained with a crystal violet solution to observe the number of migrated cells.

### 2.10 Statistical analysis

All statistical analyses were conducted using R software (version R-4.1.0) and GraphPad Prism 8.0.2. The Wilcoxon test was used for statistical analysis between two groups, and the Kruskal—Wallis test was selected flexibly when there were three or more groups. Spearman correlation analysis was used for bivariate correlation analysis. The significance level is denoted as follows: *p < .05, **p < .01, ***p < .001.

## 3 Results

### 3.1 Identification of differentially expressed cellular senescence-related genes in HCC

**FIGURE 1:**
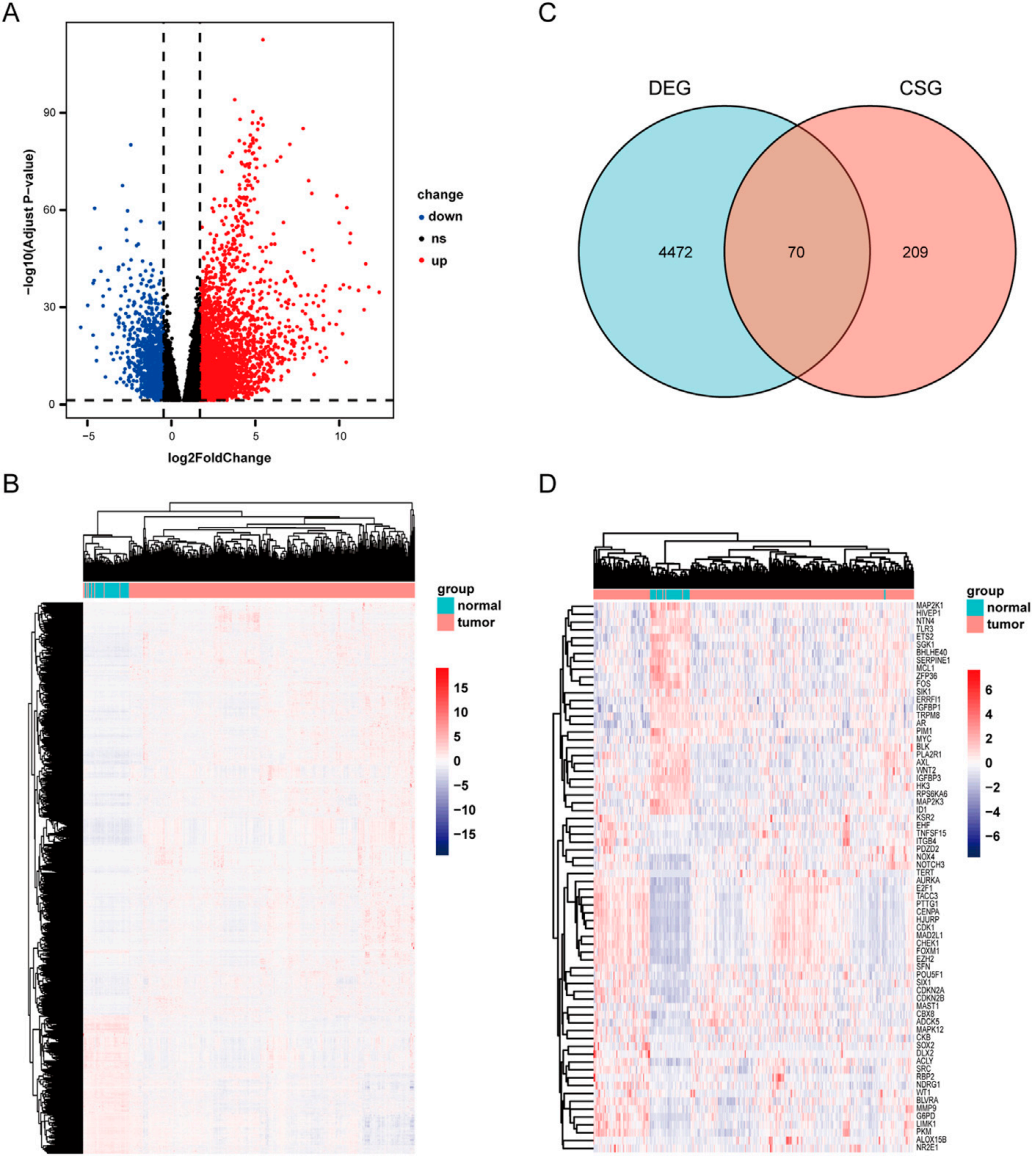
Scanning differentially expressed celluar senescence-related DEGs(CSGs). **(A)** Differentially expressed genes in TCGA dataset. |LogFC|>1 and adj. *p*-value <.05 were set to screen. **(B)**Heatmap of the differentially expressed genes in TCGA dataset. **(C)** Venn diagram representing the intersection of DEGs and celluar senescence-related genes. **(D)** Heatmap of the CSGs in TCGA dataset.

Through analyzing the DEG profiles of 369 HCC samples and 50 adjacent cancer samples from the TCGA database, 3,352 upregulated DEGs and 1,190 downregulated DEGs were obtained (Supplementary Table S1), which are shown in Figures 1A, B. Then, by intersecting the above 4,542 DEGs with 279 cellular senescence-related genes, 70 CSGs were identified (Figure 1C), which are shown in Figure 1D.

### 3.2 Construction of a prognostic model based on cellular senescence-related genes

**FIGURE 2:**
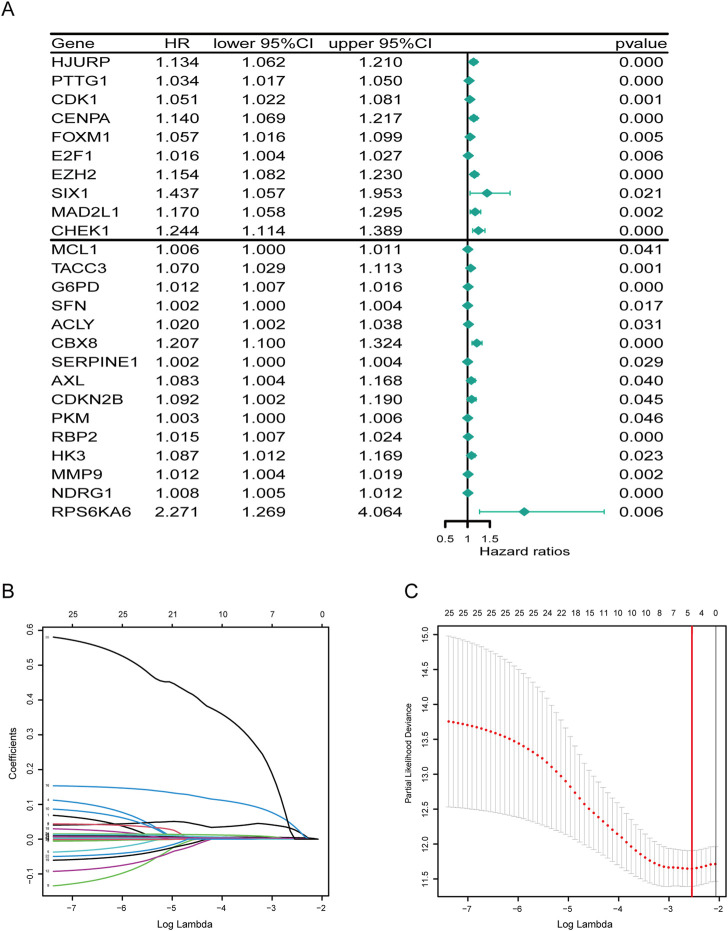
Developing a prognostic signature. **(A)** Forest plot of 25 cellular senescence-related genes associated with HCC prognosis. **(B)** The coefficients in the LASSO regression model for CSGs. **(C)** A minimum value of λ was chosen as optimal. The red linerepresents those 25 features that were reduced to four non-zero coefficient features by LASSO.

**FIGURE 3:**
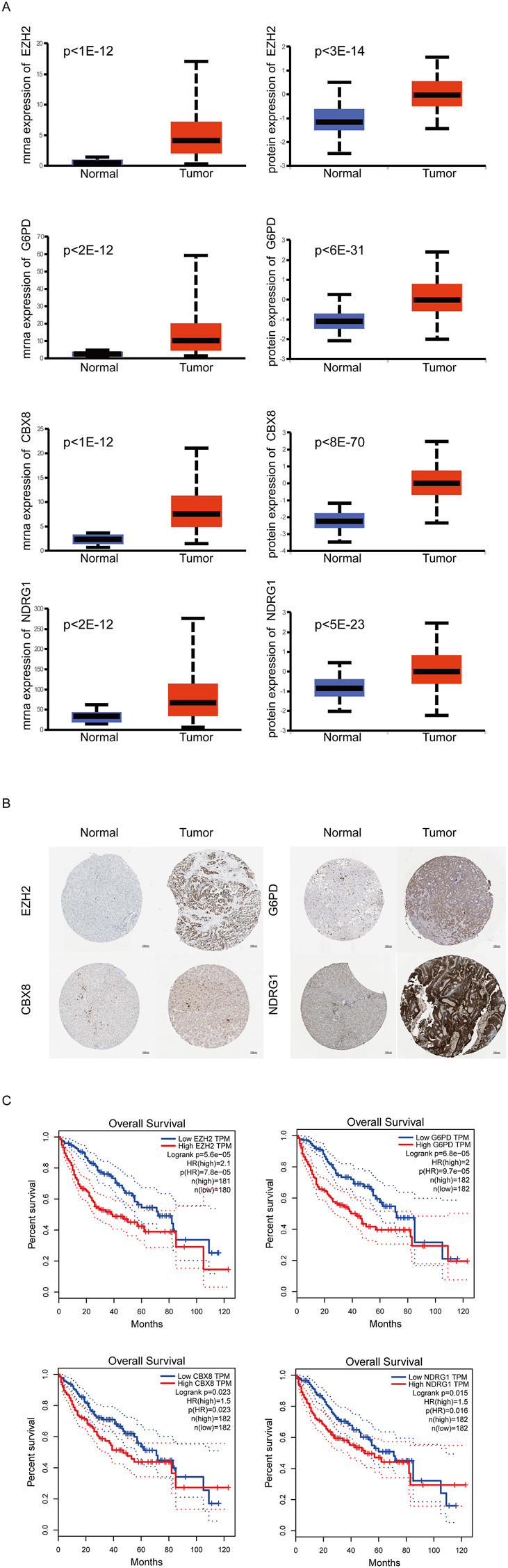
Expression of Key CSGs in HCC. **(A)**UALCAN analysis of 4 CSGs.**(B)** IHC of 4 CSGs between liver cancer specimens and normal specimens in HPA dataset. **(C)** Overall survival (OS) curves of 4 CSGs in HCC patients.

Next, through univariate cox analysis of the above 70 CSGs, 25 genes were identified to be associated with HCC prognosis (*p* < .05) (Figure 2A) (Supplementary Table S2). Then, to avoid excessive variables which may result in overfitting, we performed the least absolute shrinkage and selection operator (LASSO) Cox regression analysis to narrow down the above 25 genes, and identified a total of four genes (EZH2, G6PD, CBX8, NDRG1) (Figures 2B, C). Furthermore, to explore the significance of the four genes, we analyzed the mRNA and protein levels in HCC specimens through UALCAN analysis, and found all the four genes were highly expressed in the HCC specimens, compared to those in normal specimens (*p* < .01) (Figure 3A). Consistently, IHC analysis also confirmed the high expression of the four proteins in HCC tissues analyzed by the Human Protein Atlas database (https://www.proteinatlas.org/) HPA dataset (Figure 3B). Moreover, survival curve analysis found that HCC patients with highly expressions of EZH2, G6PD, CBX8, or NDRG1 has shorter survival period (Figure 3C). Finally, we constructed a prognostic risk signature with the above four genes through LASSO algorithm, and the formula is as: risk score = (.0266358*expression of EZH2) + (.0043178*expression of G6PD) + (.0352105*expression of CBX8) + (.0019414*expression of NDRG1).

### 3.3 Evaluation of the prognostic stratified model

**FIGURE 4:**
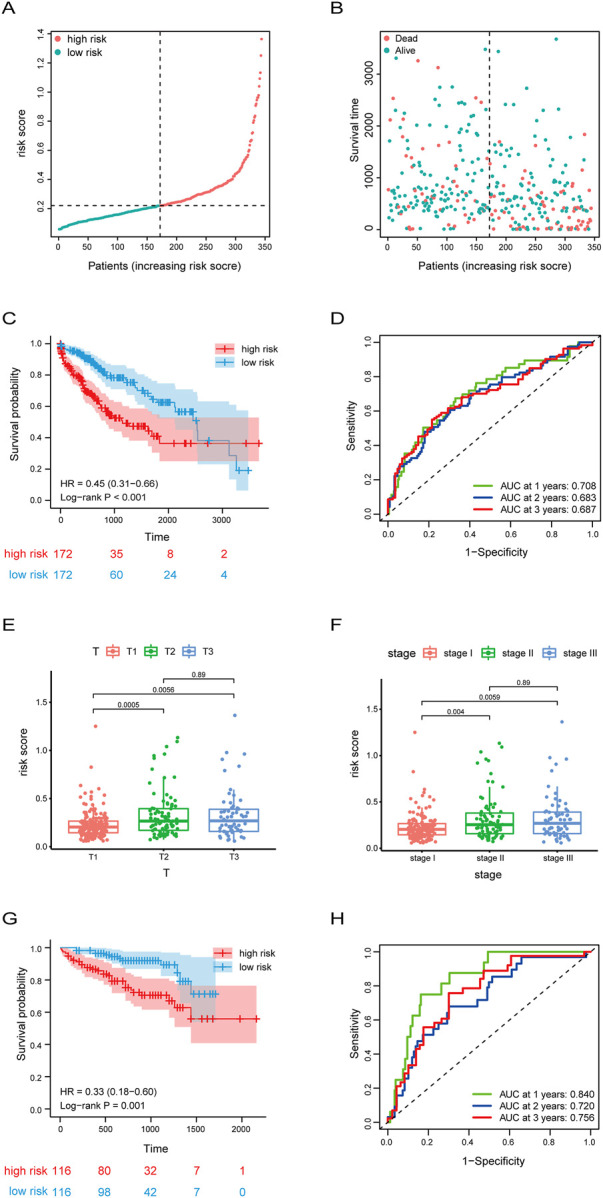
Risk score signature predicted prognosis for HCC patients. **(A,B)** The distribution of risk score, patients’ survival and status for HCC. The black dotted line divided patients into high risk group and low risk group. **(C)** Kaplan-Meier survival analysis of patients stratified by the median risk score in TCGA dataset. **(D)** The ROC curve was applied to assess the predictive 1-year, 2-year, 3-year performance of riskscore in TCGA dataset. **(E,F)**Box plot of the difference in risk score for patients with different T-stages (tumor size) and stages. **(G)** Kaplan-Meier survival analysis of patients stratified by the median risk score in ICGC dataset. **(H)** The ROC curve was applied to assess the predictive 1-year, 2-year, 3-year performance of riskscore in ICGC dataset.

To further access the prognostic risk signature, HCC patients from the TCGA dataset were stratified into two groups based on the median risk score (.2204) (Figure 4A). In ascending order of riskscore, the patient’s survival status was shown in Figure 4B. Survival probability analysis showed that HCC patients in the high-risk group have a poor prognosis, compared to the low-risk group (Figure 4C). Moreover, we used ROC curve to estimate the predictive value of our model, and found that the AUC was .708 at 1 year, .683 at 3 years, and .687 at 5 years, indicating well predictive value (Figure 4D). In addition, we analyzed the correlation between risk score and clinicopathological characteristics of HCC patients, and found that the risk scores of HCC patients in the T2 and T3 stage were significantly higher than those in the T1 stage (Figure 4E). Similarly, the risk scores of HCC patients in the stageⅡand stage Ⅲ were significantly higher than those in the stageⅠ(Figure 4F).

Subsequently, to further validate the accuracy of the prognostic stratified model, we used the model in the HCC samples of the ICGC dataset which was used as a validation set. Consistently with the results of Figure 4C, the survival probability of the high-risk group with HCC was lower (Figure 4G). In addition, the AUC of HCC patients was .84 at 1 year, .72 at 3 years, and .756 at 5 years (Figure 4H). Overall, these results suggest the good prognostic performance of our model.

### 3.4 Establishment of the nomogram for the prediction of the HCC patients’ survival probability

**FIGURE 5:**
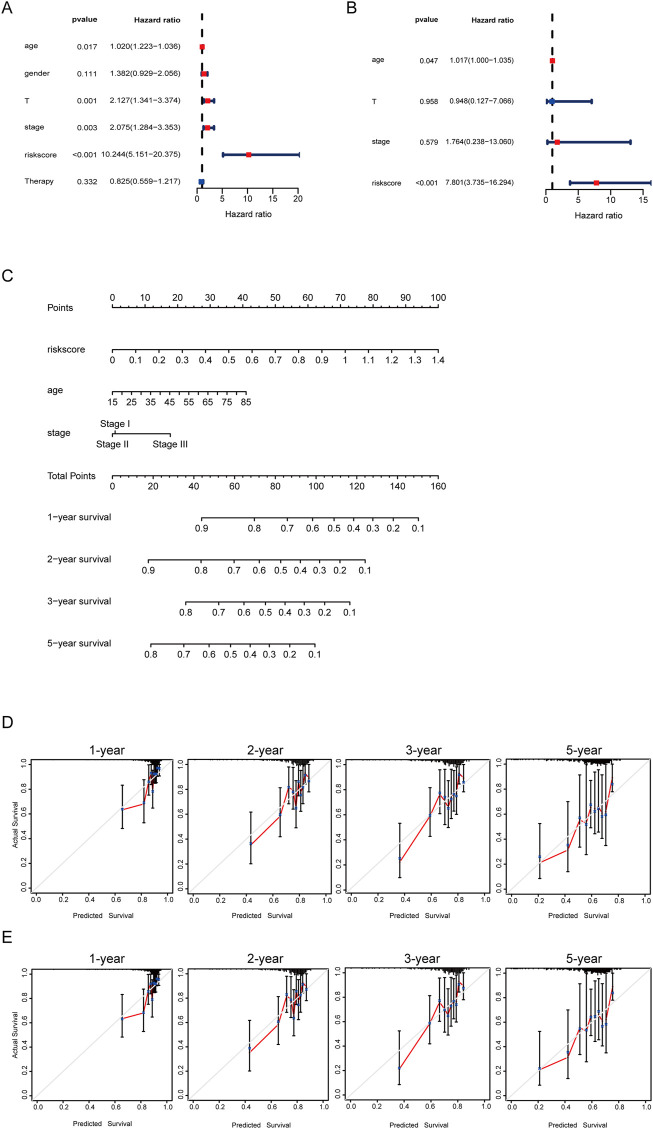
Developing a nomogram for predicting survival. **(A)** Univariate cox regression forest plot of risk score and clinical information. **(B)** Multivariate cox regression forest plot of risk score and clinical information. **(C)** Nomogram for the prediction of the HCC patients’ survival probability at 1, 2, 3, and 5 years. **(D)** Calibration curves of TCGA dataset. **(E)** Calibration curves of ICGC dataset.

Subsequently, the univariable analysis and multivariable analysis based on the age, the pathologic stage and the risk score showed the riskscore could be as an independent prognostic factor (Figures 5A, B). Furthermore, to make it easier for clinicians to predict the survival probability of HCC patients, a nomogram was constructed based on the risk score, age, pathologic stage, and survival rate (Figure 5C). The total point is calculated by adding the risk score, age and stage scores, from which the probability of survival of 1, 2, 3, and 5 years is intuitively predicted. Moreover, calibration plots showed good consistencies between the predicted survival and the actual survival at 1, 2, 3, and 5 years in the training set (Figure 5D) and in the validation set (Figure 5E).

### 3.5 Analysis of the correlation between the risk signature and genetic mutations

**FIGURE 6:**
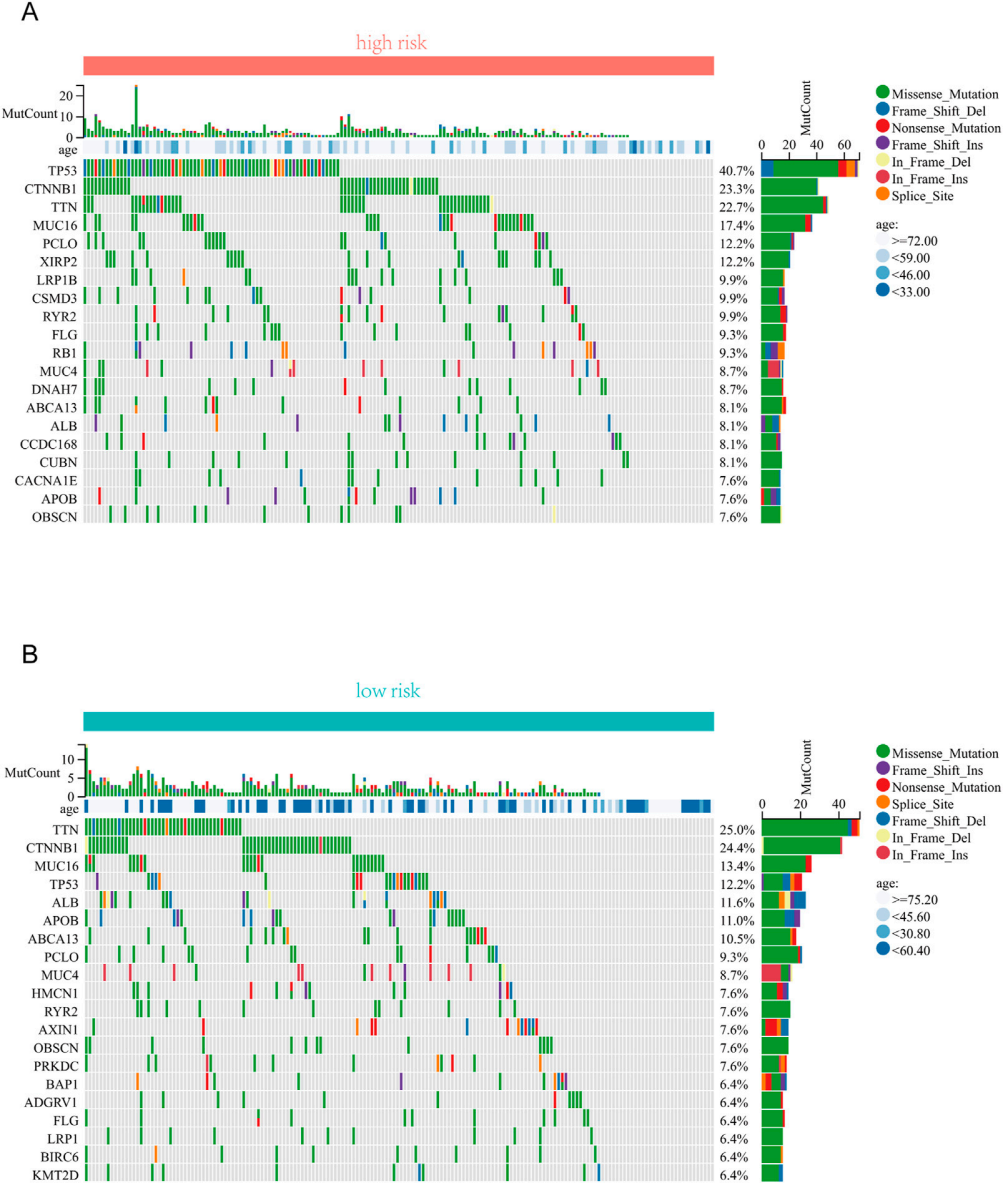
Correlation analysis between risk score and TMB . **(A)** The top 20 driver genes with the highest alteration in the high-risk group. **(B)** The top 20 driver genes with the highest alteration in the low-risk group.

We further investigated the differences in somatic mutation distribution between low and high risk scores in the TCGA set. As shown in the waterfall plot (Figures 6A, B), tumor mutational burden (TMB) differences exist in two subtypes, and the mutation frequency of TP53, MUC16, and PCLO in the high risk group was significantly higher than those the low risk group.

### 3.6 Analysis of the risk signature and immune characteristics

**FIGURE 7:**
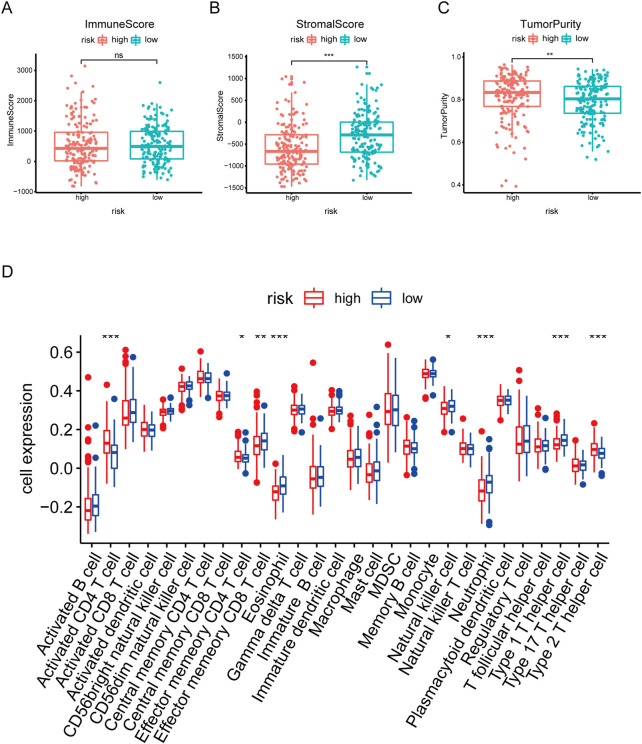
Correlation analysis between risk score and Immune infiltration in HCC. **(A–C)** Box plot of differences in immuneScore, StromalScore TumorPurity between high- and low-risk groups. **(D)** Box plot of differences in immune cell infiltration in high- and low-risk groups. *p < .05. **p < .01. ***p < .001.

Emerging studies report immune dysfunction is significantly associated with HCC development (Mizukoshi and Kaneko, 2019), and cellular senescence acts essential roles in immune dysfunction (Castelo-Branco and Soveral, 2014). To further analyze the immune characteristics of the risk signature in HCC, we investigated the associations between immune cells and risk scores. As shown in Figure 7A, there was no statistical difference of the immune score between the high risk score group and the low risk score group. However, the high risk score group had lower stromal score and higher tumor purity, compared to the low risk score group (Figures 7B, C). Furthermore, ssGSEA analysis showed that the abundance of activated CD4 T cells, effector memory CD4 T cells, and type 2 T helper cells in the high-risk group was significantly higher (Figure 7D). While the abundance of effector memory CD8 T cells, eosinophil, neutrophil, and type 1 T helper cells in the high-risk group was evidently decreased (Figure 7D). Overall, these results suggest that the tumor microenvironment and immune infiltration between the two groups are significantly different.

### 3.7 Analysis of drug sensitivity and immune checkpoint

**FIGURE 8:**
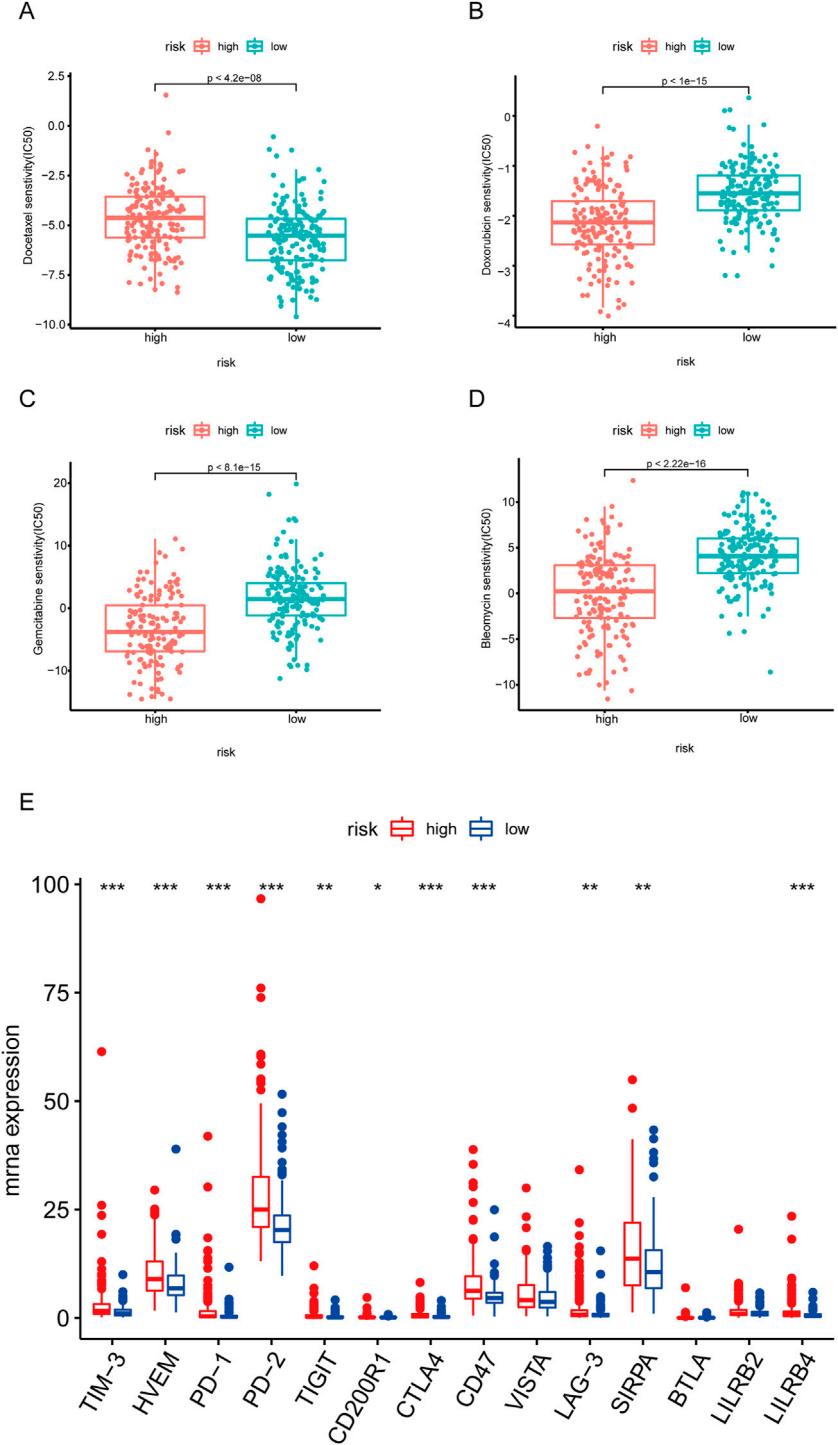
Correlation of risk score with immunotherapy. **(A–D)** Box plot of the differences in IC50 of docetaxel, doxorubicin, gemcitabine, and bleomycin between high- and low-risk groups. **(E)** Box plot of the differences in immune checkpoint between high- and low-risk groups. *p < .05. **p < .01. ***p < .001.

To investigate the sensitivity of HCC patients to traditional anti-tumor drugs, we analyzed the IC50 of anti-tumor drugs in the high and low risk score group. As shown in Figure 8A, the IC50 of docetaxel in the high-risk group was significantly higher (Figure 8A), while the IC50 of doxorubicin, gemcitabine, and bleomycin in the high-risk group were evidently lower, compared to those in the low-risk group (Figures 8B–D).

Given that immune checkpoint inhibitors have been more and more widly used for cancer therapy in clinical (Cheng et al., 2020; de Miguel and Calvo, 2020), we further investigated the expressions of immune checkpoints in the high and low risk score group. As shown in Figure 8E, the expressions of TIM-3, HVEM, PD-1, PD-2, TIGHT, CTLA4, CD47, LAG-3, and SIRPA were significantly higher in the high-risk group.

### 3.8 Functional enrichment analysis and PPI network construction

**FIGURE 9:**
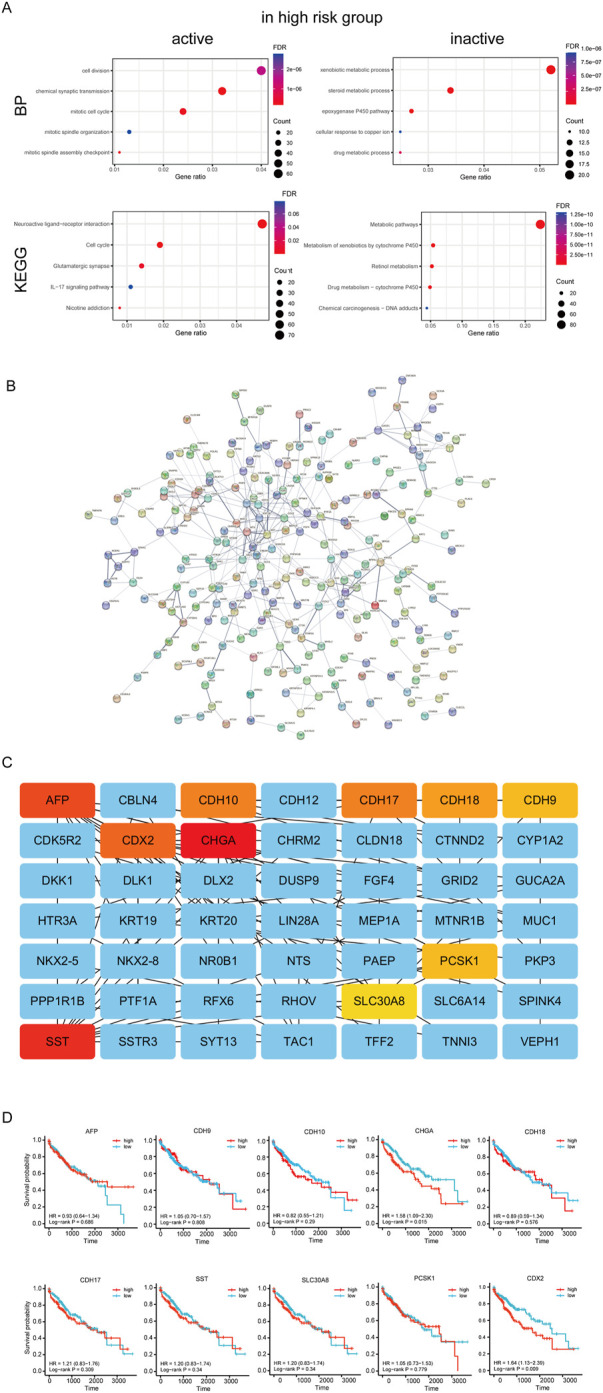
Analysis of differences between high and low risk groups. **(A)** Enrichment analysis between high and low risk groups. **(B)** PPI network of the differential expressed genes of high and low risk groups.**(C)** Hug genes of PPI network between high and low risk groups **(D)** Survival curve of hub genes.

Next, we performed GO annotation and KEGG pathway enrichment analysis to explore the potential biological functions of the DEGs between the high and low-risk groups of TCGA which were shown in Supplementary Table S3. The enriched GO annotation included cell division, chemical synaptic transmission, and mitotic cell cycle in the biological process (BP) category is active in high risk group, and xenobiotic metabolic process, steroid metabolic process and epoxygenase P450 pathway is inactive (Figure 9A). KEGG pathway enrichment analysis showed that these upregulated DEGs were mainly enriched in the neuroactive ligand-receptor interaction, cell cycle, and glutamatergic synapse and downregulated DEGs were mainly enriched in metabolic pathways, Metabolism of xenobiotics by cytochrome P450 and retinol metabolism (Figure 9A). Then, the protein-protein interaction (PPI) network of the DEGs between the high and low-risk groups was analyzed by the STRING database (Figure 9B). Furthermore, 10 hub genes (AFP, CDH10, CDH17, CDH18, CDH9, CDX2, CHGA, PCSK1, and SLC30A8SST) were identified by Cytoscape plugin cytoHubba (Figure 9C). Moreover, survival analysis revealed HCC patients with high expression of CDX2 (DNA-binding transcription factor activity and transcription corepressor activity) or CHGA (autocrine or paracrine negative modulators of the neuroendocrine system) had lower survial proability (Figure 9D).

### 3.9 CSGs promotes HCC cell migration and invasion

**FIGURE 10:**
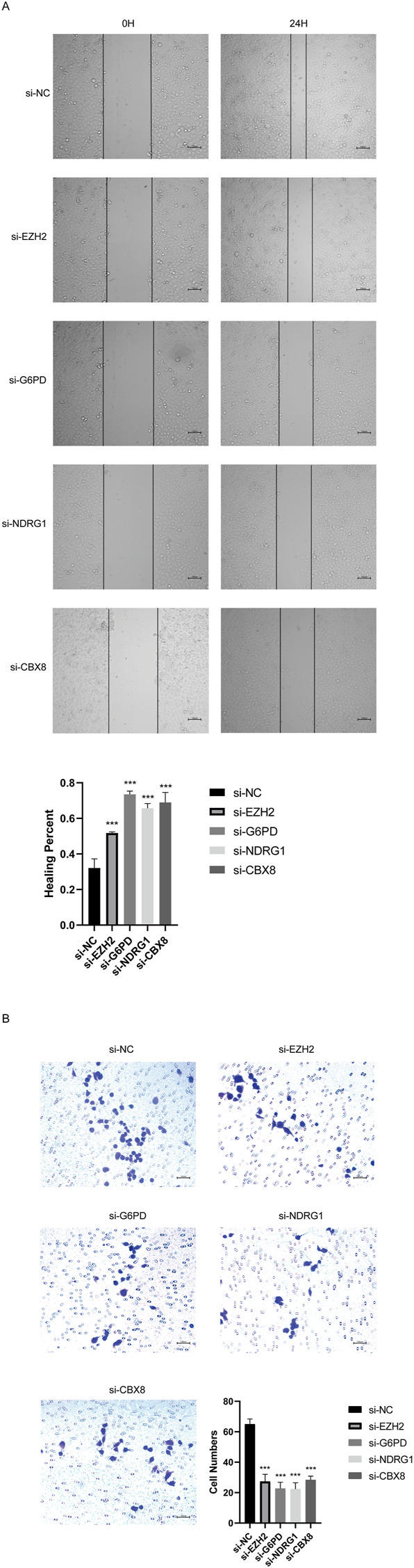
CSGs inhibits SMMC-7721 cells migration, and invasion *in vitro*. **(A)** Silencing 4CSGs attenuated wound closure corroborated in SMMC-7721 (*n* = 3). **(B)** Silencing 4 CSGs attenuated wound closure corroborated in SMMC-7721. The error bars indicate the mean ± SD, and each experiment was repeated at least three times. *p < .05, **p < .01, ***p < .001.

In order to verify the roles of 4 CSGs on HCC cell migration and invasion, these expressions were knockdown by transfection with specific siRNAs in SMMC-7721 cells. As shown in Figures 10A, B, downregulationof EZH2, G6PD, CBX8, or NDRG1 significantly inhibited the migration and invasion of SMMC7721 cells, respectively (Figures 10A, B).

## 4 Discussion

Cellular senescence is characterized by functional decline and hypometabolism, such as cell cycle arrest, loss of the ability of cells to replicate, and permanent cessation of cell division (Shih et al., 2017). In the early stages of tumors, cellular senescence is activated by oncogenes and participates in suppressing tumor development (Zeng et al., 2018). However, with the tumor progression, senescent cancer cells continue to increase, which transforms to promote tumor progression (Prieto and Baker, 2019). Therefore, cellular senescence is emerging as a novel potential anti-tumor strategy (Prasanna et al., 2021). In this study, we established an cellular senescence-related DEGs-based, accurate stratified model, and a nomogram based on multivariable analysis, whihc have potentially important roles in guiding clinical therapy for HCC.

In the present study, we used four genes (EZH2, G6PD, CBX8, and NDRG1) to construct the risk prognostic score signature in HCC. EZH2 is reported to inhibit gene transcription by methylates Lys9 and Lys27 on histone H3, and suppress cellular senescence phenotypes by inactivating p16 and p21 (Bracken et al., 2003; Fan et al., 2011; Tzatsos et al., 2011). Besides, EZH2 is highly expressed in a variety of tumors, promote tumor development by regulating cell cycle, and is related to the degree of tumor malignancy (Duan et al., 2020). Recently, EZH2 has also been reported to promote the proliferation and metastasis of HCC (Lei and Wang, 2022). Similarly, our study finds HCC patients with EZH2 high expression have low survival rates. G6PD is a rate-limiting enzyme in the pentose phosphate pathway, and its deficiency accelerates cellular senescence (Ho et al., 2000). Whereas, high expression of G6PD promotes tumor growth by generating ribo-5-phosphate and NAPDH (Stanton, 2012). G6PD is reported to induce epithelial-mesenchymal transition, thereby promoting HCC cell invasion (Lu et al., 2018). In addition, G6PD promotes HCC development by inhibiting ferroptosis through targeting cytochrome P450 oxidoreductase (Cao et al., 2021). Consistently, our study showed knockdown of G6PD evidently suppressed HCC cell migration and invasion. CBX8 inhibits stress-induced premature senescence in K562 leukemia cells by regulating AKT-RB-E2F1 pathway (Lee et al., 2016). Recent study reported CBX8 interact with YBX1 to regulate cell cycle and promote the growth of HCC cells (Xiao et al., 2019). NDRG1, a hypoxia-inducible protein, is identified as a tumor suppressor in gliomas and glioblastomas (Nakahara et al., 2022). However, NDRG1 is found to be highly expressed in HCC, and promotes hepatocellular carcinoma proliferation and metastasis (Liu et al., 2019; Dang et al., 2020). Inhibition of NDRG1 triggers senescence of HCC cells through activating glycogen synthase kinase-3β-p53 pathway (Lu et al., 2014). Knockdown of NDRG1 promotes the pro-poptotic protein BAX expression and mitochondria division in HCC cells, thereby inhibiting HCC progression (Dang et al., 2020). Consistently, Knockdown of NDRG1 notably suppressed HCC cell migration and invasion. Given the higher expression levels of EZH2, G6PD, CBX8, NDRG1 in the high risk score scroe group of HCC patients, we speculate that targeting cellular senescence to treat HCC may have a better therapeutic effects for such patients.

Nowadays, although chemotherapy has been widely used for HCC therapy in clinical, the clinical chemotherapy outcomes were not satisfied, owing to chemotherapy resistance (Lohitesh et al., 2018). Therefore, it is particularly important to screen out patients who are sensitive to chemotherapeutic drugs and then carry out individualized treatment (Zhang and Chen, 2020). Recent studies report that the aberrant expression of phosphatidylinositol 3-kinase (PI3K)/AKT signaling in HCC contributes to highly resistant to treatment with docetaxel (Wang et al., 2021). EZH2 is identified to promote the progression of osteosarcoma through activating AKT (Wan et al., 2022). In this study, we investigated sensitivity differences to chemotherapeutic agents in high- and low-risk groups of HCC patients, and found HCC patients in the low-risk group were more susceptible to Docetaxel. This is consistent with the lower EZH2 expression in the low-risk groups of HCC patients. Interestingly, the high-risk groups of HCC patients were more sensetive to doxorubicin, gemcitabine, and bleomycin, which provides a benificial guide for precise treatment in clinical. However, the underlying mechanism needs further investigation. Immunotherapy, particularly immune checkpoint inhibitors, has achieved initial success in the treatment of various cancers, including HCC (Wen et al., 2022). Commonly, the efficacy of immunotherapy largely depends on the tumor immune microenvironment (Miao and Nan, 2022). In our stratified model, higher infiltration levels of effector memory CD8 T cells and NK cells, lower infiltration levels of type 2 helper T cells were identified in the low-risk group of HCC, compared to those in the high-risk group of HCC. Meanwhile most of the immune checkpoints in high-risk patients with HCC were upregulated, including CD8 (+) T-cell immune checkpoints (PD-1, CTLA-4, and TIGIT) and NK cell immune checkpoints (PD-1, LAG-3, and TIM-3) (Zhou et al., 2019). These findings suggest that HCC patients with high risk prognostic scores may be more suitable for thearpy with immune checkpoint inhibitors.

KEGG analysis showed the differential genes between the high and low risk group of HCC were mainly enriched in the neuroactiveligand-receptor interaction pathway. Recently, high expressions of genes involved in the neuroactive ligand-recepotr interaction pathway is found to be associated with poor prognosis in papillary renal cell carcinoma (Li et al., 2022). Similarly, in our study, HCC patients in the high risk score group with shorter survival rates also have higher expressions of the neuroactive ligand-recepotr interaction pathway-related genes. Furthermore, PPI network analysis showed ten hub genes (AFP, CDH10, CDH17, CDH18, CDH9, CDX2, CHGA, PCSK1, SLC30A8, and SST), and HCC patients with high expression of CDX2 and CHGA had poor prognosis. CDX2, known as a nuclear transcription factor, plays important roles in regulating the epithelial to mesenchymal transition, and acts as a prognostic factor and emerging biomarker in colon cancer (Grainger et al., 2010; Dalerba et al., 2016). A pathological staining survey found that poorly differentiated HCC had more CDX2 expression (Zhou et al., 2019), which also indicated that patients in the high-risk group had poorly differentiated HCC. CHGA, known as Chromogranin A (CgA), a protein stored in the secretory granules of many neuroendocrine cells and neurons, could be detected in the blood of patients with neuroendocrine tumors or heart failure (Seregni et al., 2001; Ferrero et al., 2004) CHGA could also be detected in the serum of some HCC patients (Leone et al., 2002). However, the detailed role of CHGA in HCC development needs further investigation in the following studies.

## 5 Conclusion

In conclusion, the prognostic signature based on cell senescence constructed in this study are helpful to predict the survival of HCC and guide clinical treatment. It is found that patients in high-risk group are more tolerant to chemotherapy drugs, but more suitable for immunotherapy. However, more experiments and clinical cases are needed to validate these findings.

## Data Availability

The original contributions presented in the study are included in the article/Supplementary Material, further inquiries can be directed to the corresponding authors.
